# Iron-associated central macular ganglion cell complex thinning and choroidal vascularity index elevation in transfusion-dependent β-thalassemia: potential OCT/OCTA biomarkers

**DOI:** 10.1186/s12886-025-04510-0

**Published:** 2025-12-12

**Authors:** Ningfeng Li, Liang Liang, Wenzhi Huang, Wenwen Li, Quanwen Zhao, Guiling Zhao, Yunxia Leng, Danna Chen

**Affiliations:** 1 Department of Ophthalmology, Guangzhou First People’s Hospital, The Second Affiliated Hospital, School of Medicine, South China University of Technology, Guangzhou First People’s Hospital, Guangzhou Medical University, Guangzhou, 510180 China; 2 Department of Ophthalmology, Affiliated Hospital of Guangdong Medical University, Zhanjiang, 524000 China

**Keywords:** Transfusion-dependent β-thalassemia, Optical coherence tomography angiography, Macular thickness, Choroidal vascular index, Iron overload

## Abstract

**Background:**

This study aimed to evaluate alterations in macular microstructure and blood flow density in patients with transfusion-dependent β-thalassemia (TDT) using optical coherence tomography (OCT) and optical coherence tomography angiography (OCTA), and to explore their associations with markers of iron metabolism.

**Methods:**

A cross-sectional study was conducted involving 31 TDT patients (61 eyes) and 31 age- and sex-matched healthy controls (61 eyes) at the Affiliated Hospital of Guangdong Medical University from October 2022 to December 2023. Data collected included basic information, medical history, best-corrected visual acuity, ocular biometry, fundus photography, and OCT/OCTA imaging. Parameters assessed comprised the thickness and blood flow density of individual macular layers, as well as choroidal stromal area, choroidal luminal area, and choroidal vascular index (CVI). Pearson correlation analysis was employed to examine relationships among macular parameters, serum ferritin (SF) levels, duration of iron chelation therapy (ICT), and other variables.

**Results:**

Compared to healthy controls, TDT patients exhibited significant thinning of the central macular area ganglion cell layer, retinal nerve fiber layer, and ganglion cell complex (mGCC), accompanied by increased blood flow density in the deep vascular complex, nerve fiber layer vascular plexus, and deep capillary plexus (all *P* < 0.05). The choroidal stromal area was reduced (*P* = 0.037), whereas CVI was elevated (*P* = 0.034). Pearson correlation analysis revealed a positive correlation between central mGCC thickness and the duration of ICT (*r* = 0.280, *P <* 0.05),and a significant positive correlation between CVI with SF levels (*r* = 0.426, *P* < 0.01). Stepwise linear regression analysis identified the duration of ICT (*β* = 0.306, *P* = 0.011) as a significant independent factor influencing central mGCC thickness. Similarly, SF levels significantly influenced CVI (*β* = 0.469, *P* < 0.001). Additionally, 62.30% of TDT patients’ eyes presented with tilted optic discs, 34.43% showed tessellated fundus, and 6.56% had pseudoxanthoma elasticum.

**Conclusions:**

TDT patients exhibit both macular neuroretinal thinning and increased microvascular blood flow density. CVI and central mGCC thickness may serve as potential biomarkers for monitoring iron-related ocular pathology and therapeutic efficacy. Future longitudinal studies are warranted to validate their predictive value and establish causality. Regular ocular screening is warranted in this population.

**Supplementary Information:**

The online version contains supplementary material available at 10.1186/s12886-025-04510-0.

## Background

Transfusion-dependent β-thalassemia (TDT), a hereditary hemolytic disorder caused by β-globin gene mutations, necessitates lifelong blood transfusions for survival. However, chronic iron overload secondary to transfusions leads to multi-organ damage, including hepatic, cardiac, and endocrine dysfunction [1–3]. It is recognized globally as a significant public health challenge [4]. Studies have demonstrated that approximately 46.87% of TDT patients exhibit ocular complications, including retinal thinning, pseudoxanthoma elasticum (PXE) changes, and microstructural abnormalities in the macular region, all of which significantly impair patients’ quality of life [5–7]. In recent years, the cumulative effect of regular blood transfusions and iron overload on the eyes has gained increasing attention, but the specific mechanism of its influence on macular microstructure and hemodynamics remains unclear.

The macula, as the vision-sensitive core of the retina, relies on the structural integrity of layers including the ganglion cell complex (GCC) and photoreceptor layers. Chronic anemia and iron overload in TDT patients induce oxidative stress and mitochondrial dysfunction, triggering retinal ganglion cell (RGC) apoptosis and vascular endothelial damage [7–10]. Evidence suggests significant macular thinning and microstructural disruptions in TDT patients [6, 11], yet comprehensive analyses integrating full-thickness retinal parameters with hemodynamic metrics are lacking. Optical coherence tomography (OCT) and angiography (OCTA) provide non-invasive, high-resolution quantification of retinal layers and microvascular density, offering novel insights into disease progression. Previous OCT/OCTA studies have mostly focused on a single structural parameter [12–14]. However, the changes in the structure of each layer in the macular area and blood flow density, as well as their correlations with iron metabolism indicators, have not been systematically clarified.

Furthermore, the southern part of China is a high-incidence area of thalassemia. The carrying rates of thalassemia in Guangxi and Guangdong are as high as 24.5% and 17.7% [15–17], respectively. This region-specific genetic burden provides a unique population for investigating iron overload-induced ocular complications. However, up to now, the epidemiological data of characteristic fundus changes in the TDT patient population are still limited.

This study aims to systematically characterize macular structural and blood flow density changes in a Southern Chinese TDT cohort using OCT/OCTA, while elucidating their correlations with iron overload indicators and therapeutic histories. By identifying biomarkers of retinal iron toxicity, this research seeks to optimize screening protocols and inform targeted interventions for TDT-associated retinopathy.

## Methods

### Study population

This cross-sectional study was conducted from October 2022 to December 2023. A total of 31 patients diagnosed with TDT and managed in the Department of Hematology of Guangdong Medical University Affiliated Hospital were enrolled (TDT group), along with 31 age- and sex-matched healthy children (control group).

Inclusion criteria for the TDT group were as follows: According to the “Chinese guideline for diagnosis and treatment of transfusion dependent β-thalassemia (2022)” [2], patients aged 4–18 years diagnosed with TDT by two deputy directors/chief physicians of the Department of Hematology at the Affiliated Hospital of Guangdong Medical University, who had received regular and standardized erythrocyte replacement therapy for ≥ 1 year and had taken iron chelating agents regularly for ≥ 3 months, and had stable vital signs. The control group comprised healthy children matched by age and gender during the same period.

The exclusion criteria were as follows: (1) Patients with other types of anemia or other significant systemic diseases; (2) History of ocular surgery (including laser treatment) for conditions such as glaucoma, corneal disease, vitreous disease, or retinal disease; (3) History of ocular trauma, congenital ocular diseases, or contact lens wear; (4) Axial length (AL) > 26 mm, spherical equivalent refraction (SER) ≤ − 6.00 diopters (D), SER ≥ + 3.00 D, or astigmatism ≥ + 2.00 D; (5) Inability to cooperate sufficiently to complete ophthalmic examinations or poor image quality due to poor cooperation. Before the study began, the researchers provided a comprehensive explanation to all the guardians of the patients and clarified the purpose of the study and possible discomforts. Written informed consent was obtained from all guardians, and assent was obtained from participants where appropriate, ensuring their voluntary participation. This study adheres to strict privacy protection measures to safeguard the personal information of all participants and their guardians. This study was approved by the Clinical Research Ethics Committee of the Affiliated Hospital of Guangdong Medical University (approval number: KT2023-016-01). All procedures involving human participants were performed in accordance with the ethical standards of this committee and with the 1964 Helsinki Declaration and its later amendments.

### Measurements

Basic information for all subjects was collected, including name, age, gender, height, past medical history and weight data. And calculate the body mass index (BMI). For the TDT group, the following data were collected: blood transfusion duration, pre-transfusion hemoglobin (Hb) level, SF level within the past month, and the types and duration of iron chelation therapy (ICT). All subjects underwent standardized ophthalmic examinations such as best-corrected visual acuity (BCVA), refraction examination, slit lamp examination, fundus examination, and non-contact intraocular pressure (IOP) measurement. The BCVA was examined using the national standard logarithmic visual acuity chart (GB11533-1989). Laser scanning fundus photography was performed using the Optos Daytona P200T (Optos plc, Dunfermline, UK). AL was measured using the Lenstar LS900 ocular biometer (HAAG-STREIT, Koeniz, Switzerland). The refraction was examined after ciliary muscle paralysis using a computerized automatic refractometer (CRK-8800, CHAROPS, South Korea), and the spherical equivalent refraction (SER) was calculated. Each eye was measured three times consecutively, and the average value was recorded. All the examiners were optometrists or ophthalmologists who have received training from the Affiliated Hospital of Guangdong Medical University.

Spectral-domain optical coherence tomography (SD-OCT) and OCT angiography (OCTA) imaging were performed for all participants using the SPECTRALIS^®^ OCT device (Heidelberg Engineering GmbH, Heidelberg, Germany). OCT scanning was used to obtain the images of the retinal structures of each layer in the macular area with the fovea centralis as the center and the scanning range being 9 mm×9 mm. The macula was divided according to the Early Treatment Diabetic Retinopathy Study (ETDRS) grid sectors: a central circular zone (1 mm diameter), an inner subfield (3 mm diameter minus central zone), and an outer subfield (6 mm diameter minus inner and central zones) (Supplementary Figure Sa). The inner and outer subfields were further subdivided into superior, inferior, nasal, and temporal quadrants, resulting in nine sectors in total. The thickness of each quadrant of each layer of the macular retina was measured using the built-in image analysis of the software. Enhanced depth imaging (EDI) mode was used to acquire a B-scan centered on the fovea for choroidal analysis, with a measurement window width of 1500 μm positioned beneath the fovea (Supplementary Fig. Sb) [18].

OCTA scans were acquired over a 3 × 3 mm area centered on the fovea for the blood vessels of each layer of the retina and choroid. Vascular parameters assessed included vessel density in the following plexuses: the macular retina, superficial vascular complex (SVC), deep vascular complex (DVC), nerve fiber layer vascular plexus (NFLVP), superficial vascular plexus (SVP), intermediate capillary plexus (ICP), deep capillary plexus (DCP), avascular complex, choriocapillaris, choroid, and full of the above layers. The original OCTA images were exported and processed using ImageJ software (version 1.53; National Institutes of Health, Bethesda, MD, USA). Images were converted to 8-bit grayscale format and binarized using the Niblack auto-local thresholding method [19] to facilitate vessel segmentation. Vessel density (VD) was then calculated within the specified regions as the percentage area occupied by vessels.

Choroidal vascular parameters were analyzed by ImageJ software (version 1.53). The choroid beneath the fovea within a 1500 μm width was segmented. The Niblack auto-local thresholding method [19] was applied to binarize the image, differentiating the choroidal vascular lumen (hyporeflective) from the stroma (hyperreflective) (Supplementary Fig. Sa). The luminal area, stromal area, and total choroidal area of the 1500 μm wide region of the choroid below the fovea macula of the choroid were obtained. And calculate the choroidal vascular index (CVI). All OCT and OCTA measurements were performed by a single, trained technician. To assess intra-observer reliability, a random subset of 20 scans was re-measured by the same technician one week later. The intraclass correlation coefficient (ICC) for key parameters (e.g., central mGCC thickness, CVI) exceeded 0.90, indicating excellent reproducibility.

**Definitions**

1. SER Calculation: SER = Sphere power (D) + 0.5 × Cylinder power (D).
2. BMI Calculation: BMI (kg/m²) = Weight (kg) ÷ Height² (m²).
3. mGCC Definition: The sum of the thickness of macular retinal nerve fiber layer (RNFL), ganglion cell layer (GCL), and inner plexiform layer (IPL) thicknesses.
4. Choroidal Parameters: Total choroidal area (TCA) = Choroidal luminal area (CLA) + Choroidal stromal area (CSA). CVI = (CLA / TCA) × 100%.

### Statistical analysis

The OCT/OCTA parameters were processed and calculated through the Image J (1.53) software. The data were analyzed by the Statistical Package for Social Sciences (Version 27 for Windows; SPSS Inc, Chicago, IL, USA). Categorical variables were tested using the chi-square test. Continuous variables were expressed as mean ± standard deviation (SD) or median (inter quartile range, IQR) based on data distribution, and normal distribution analysis was performed using the Kolmogorov-Smirnov test. We employed Student’s t-test and Non-parametric test for normally and abnormally distributed data, respectively. The correlation was evaluated by Pearson correlation analysis. The closer the absolute value of r was to 1, the stronger the correlation. When |r| ≥ 0.8, it was a very strong correlation. When 0.6 ≤ |r| < 0.8, it is a strong correlation. When 0.4 ≤ |r| < 0.6, it is moderately correlated. When 0.2 ≤ |r| < 0.4, it is weakly correlated. When 0 ≤ |r| < 0.2, it is a very weak correlation. *P* < 0.05 was considered statistically significant. A post-hoc power analysis was performed using PASS 15.0 software (NCSS, LLC) to assess the statistical power of the significant findings reported in this study, based on the observed effect sizes and the present sample size. A power value greater than 0.80 was considered adequate.

## Results

### Demographic and clinical characteristics

Initially, 31 TDT patients (62 eyes) and 31 healthy controls (62 eyes) were enrolled. After applying exclusion criteria (one eye from the TDT group and one from the control group were excluded, as noted in the Methods), a final dataset of 61 eyes per group (122 eyes total) was included for analysis. The TDT group comprised 31 patients (61 eyes), with 60.66% of eyes (37/61) belonging to male subjects. The mean age of TDT group is 10.31 ± 3.67 years. The control group included 31 healthy subjects (61 eyes), with males’ eye representing 50.82% (31 eyes). The mean age of control group is 11.00 ± 3.18 years. No statistically significant differences were observed between the two groups in age (*P* = 0.271) or gender (*P* = 0.274). The mean SER was − 0.39 ± 2.19 D in the TDT group and − 0.72 ± 1.29 D in the control group. There was no significant differences between the two groups (*P* = 0.323). However, the TDT group exhibited significantly shorter axial lengths compared to the control group (23.32 ± 0.97 mm vs. 24.06 ± 0.99 mm, *P* < 0.001) (Table 1). Among TDT patients, the mean values of BMI, pre-transfusion Hb level, transfusion duration, SF level (within the past month), and ICT duration were 15.39 ± 1.46 kg/m², 80.45 ± 15.96 g/L, 8.78 ± 3.79 years, 4070.00 (3024.00, 6270.00) µg/L, and 3.17 ± 2.54 years, respectively. Among the sixty-one eyes in the TDT group, the corresponding patients were receiving deferasirox (DFX) monotherapy for thirty-one eyes (50.82%) and combined DFX and deferoxamine (DFO) therapy for thirty eyes (49.18%) (Supplementary Table S1).

### Macular structural analysis

The retinal structures of each layer in the macular area were scanned by OCT and their thicknesses were analyzed. The results are detailed in Table 1. The average thickness of inner nasal, inner superior, inner temporal, outer inferior and central GCL in the macular area of the TDT group was significantly thinner than that of the corresponding structural layer thickness in the control group (*P* values were < 0.001, 0.019, < 0.001, 0.030, 0.033, respectively). The average thickness of inner nasal, inner superior, inner temporal and central GCC in the macular area of the TDT group was significantly thinner than the corresponding layer in the control group (*P* values were < 0.001, 0.039, < 0.001, 0.007, respectively). The average thickness of the macular central RNFL of the TDT group was significantly thinner than the control group (*P* = 0.022). There was no statistically significant difference in the thickness of other retinal structural layers in the macular area in the TDT group (all *P* values were > 0.05) (Table 1).

**Table 1:** Thickness difference of retinal GCL, IPL, RNFL, and GCC in macular region

Retinal location		TDT Group (*N* = 61)	Control Group (*N* = 61)	t	P ^a^
		Mean ± SD (um)			
IN	GCL	49.60 ± 6.35	53.36 ± 4.44	-3.802	**< 0.001**
	IPL	33.57 ± 11.22	36.80 ± 11.83	-1.546	0.125
	RNFL	26.87 ± 10.95	28.10 ± 11.25	-0.612	0.542
	GCC	110.03 ± 11.98	118.43 ± 8.52	-4.460	**< 0.001**
IS	GCL	51.59 ± 5.35	53.57 ± 3.72	-2.378	**0.019**
	IPL	34.74 ± 10.05	36.52 ± 9.87	-0.991	0.324
	RNFL	29.25 ± 9.04	30.33 ± 9.22	-0.654	0.514
	GCC	115.57 ± 11.18	119.61 ± 10.09	-2.092	**0.039**
IT	GCL	43.25 ± 5.63	46.66 ± 5.33	-3.435	**< 0.001**
	IPL	31.97 ± 11.64	35.30 ± 14.00	-1.428	0.156
	RNFL	24.69 ± 12.29	24.62 ± 12.54	0.029	0.977
	GCC	99.90 ± 9.82	106.33 ± 8.04	-3.955	**< 0.001**
II	GCL	50.49 ± 5.56	52.33 ± 6.84	-1.628	0.096
	IPL	34.89 ± 8.55	36.33 ± 9.57	-0.878	0.382
	RNFL	29.31 ± 9.08	29.82 ± 9.12	-0.308	0.758
	GCC	114.69 ± 11.41	117.82 ± 13.89	-1.361	0.176
ON	GCL	38.52 ± 6.56	38.52 ± 5.52	0.000	1.000
	IPL	36.98 ± 5.90	38.11 ± 6.70	-0.989	0.324
	RNFL	43.66 ± 10.71	43.05 ± 10.36	0.318	0.751
	GCC	119.16 ± 8.64	119.70 ± 9.85	-0.322	0.748
OS	GCL	35.72 ± 3.61	36.00 ± 3.23	-0.450	0.654
	IPL	31.48 ± 4.37	31.80 ± 4.25	-0.420	0.675
	RNFL	36.26 ± 6.28	35.95 ± 5.58	0.290	0.773
	GCC	103.46 ± 9.03	103.41 ± 6.99	0.034	0.973
OT	GCL	36.87 ± 4.69	37.52 ± 4.08	-0.825	0.411
	IPL	28.70 ± 7.63	29.08 ± 7.87	-0.269	0.789
	RNFL	24.15 ± 8.32	24.62 ± 9.05	-0.302	0.763
	GCC	89.72 ± 8.68	90.93 ± 9.14	-0.752	0.454
OI	GCL	33.80 ± 3.25	35.07 ± 3.11	-2.192	**0.030**
	IPL	32.69 ± 10.85	32.23 ± 5.78	0.292	0.771
	RNFL	37.18 ± 7.06	36.39 ± 6.45	0.643	0.521
	GCC	103.67 ± 10.64	103.69 ± 7.07	-0.010	0.992
Central	GCL	14.95 ± 6.49	17.49 ± 6.50	-2.161	**0.033**
	IPL	17.00 ± 6.47	18.92 ± 6.32	-1.656	0.100
	RNFL	13.11 ± 3.87	15.03 ± 5.18	-2.316	**0.022**
	GCC	45.07 ± 12.80	51.44 ± 13.06	-2.723	**0.007**

N number, IN inner nasal, IS inner superiorm, IT inner temporal, II inner inferior, ON outer nasal, OS outer superior, OT outer temporal, OI outer inferior

^a^ Continuous variables were calculated by T-test

Bold values represent significance (*p* < 0.05)

### Macular blood flow density

The blood flow density of each layer of the retina in the macular area was assessed by OCTA. The results in Table 2 show that compared with the control group, the blood flow densities of DVC, DCP and NFLVP in the TDT group were significantly increased (*P* values were 0.044, < 0.001, 0.008 in sequence), while there were no significant differences in the blood flow densities of the other structural layers (all *P* values > 0.05).

**Table 2:** The variation of blood flow density within the macular region

	TDT Group (*N* = 61)	Control Group (*N* = 61)	t	*P* ^a^
	Mean ± SD			
Full (%)	49.25 ± 23.14	50.01 ± 19.24	-0.211	0.833
Ret (%)	17.31 ± 8.40	18.36 ± 8.69	-0.679	0.499
SVC (%)	21.29 ± 8.98	19.44 ± 9.54	1.105	0.271
DVC (%)	20.33 ± 10.42	16.87 ± 8.19	2.042	**0.044**
NFLVP (%)	2.93 ± 2.12	1.72 ± 1.56	3.599	**< 0.001**
SVP (%)	30.28 ± 10.58	29.61 ± 10.97	0.342	0.733
ICP (%)	13.48 ± 7.22	13.22 ± 5.96	0.215	0.830
DCP (%)	22.96 ± 9.35	18.81 ± 7.54	2.700	**0.008**
Avascular complex(%)	3.60 ± 10.55	1.14 ± 2.34	1.778	0.080
Choriocapillaris (%)	26.73 ± 19.79	29.42 ± 12.47	-0.900	0.370
Choroid (%)	36.31 ± 18.50	36.25 ± 17.63	0.017	0.987

Full full of the above layers, Ret retina

^a^ Continuous variables were calculated by T-test

Bold values represent significance (*p* < 0.05)

### Choroidal parameters

The choroidal layer structure was scanned by OCT and OCTA, and the CSA, CLA and CVI values were calculated. The results in Table 3 show that compared with the control group, the TDT group exhibited a significant reduction in CSA (*P* = 0.037) and elevated CVI (*P* = 0.034), while choroidal CLA remained unchanged (*P* = 0.191) (Table 3).

**Table 3:** The differences in CSA, CLA and CVI between the two groups

	TDT Group (*N* = 61)	Control Group (*N* = 61)	t	*P* ^a^
	Mean ± SD			
CSA (um^2^)	0.40 ± 0.12	0.44 ± 0.10	-2.112	**0.037**
CLA (um^2^)	0.77 ± 0.18	0.82 ± 0.18	-1.315	0.191
CVI (%)	66.51 ± 3.71	62.84 ± 12.74	2.162	**0.034**

^a^ Continuous variables were calculated by T-test

Bold values represent significance (*p* < 0.05)

### Correlation and regression analysis

Pearson correlation analysis (Supplementary Table S2) revealed that central GCC thickness was positively correlated with SER (*r* = 0.336, *P* < 0.01) and the duration of ICT (*r* = 0.280, *P* < 0.05). The CVI exhibited a significant positive correlation with SF levels (*r* = 0.426, *P* < 0.01). Stepwise linear regression analysis identified SER (*β* = 0.358, *P* = 0.003) and the duration of ICT (*β* = 0.306, *P* = 0.011) as significant independent factors influencing central GCC thickness, with SER demonstrating a stronger effect (Table 4). Similarly, transfusion duration (*β* = − 0.257, *P* = 0.030) and SF levels (*β* = 0.469, *P* < 0.001) significantly influenced CVI, with SF showing a more pronounced association (Table 5). However, since no significant correlation was observed between CVI and transfusion duration (*r* = − 0.177, *P* > 0.05) (Supplementary Table S2), the clinical significance of transfusion duration on CVI requires further validation.

**Table 4:** Analysis of influencing factors of central macular GCC in the macular area of the TDT group

	Unstandardized Coefficient		Standardized Coefficient	t	*P*	VIF
	B	SE	β			
Constant	40.999	2.390		17.157	**< 0.001**	
SER(D)	2.094	0.685	0.358	3.055	**0.003**	1.006
ICT Duration(ys)	1.543	0.591	0.306	2.612	**0.011**	1.006
R^2^			0.206			
Adjusted R^2^			0.179			
F			*F* = 7.529, ***P =*** **0.001**			
D-W			1.965			

SE standard error, D diopter

Bold values represent significance (*p* < 0.05)

**Table 5:** Analysis of influencing factors of CVI in the TDT group

	Unstandardized Coefficient		Standardized Coefficient	t	*P*	VIF
	B	SE	β			
Constant	67.969	1.068		63.638	**< 0.001**	
Transfusion Duration(ys)	-0.252	0.113	-0.257	-2.222	**0.030**	1.030
SF (< 1month) (ug/L)	0.000	0.000	0.469	4.056	**< 0.001**	1.030
R^2^			0.245			
Adjusted R^2^			0.219			
F			*F* = 9.435, ***P*** **< 0.001**			
D-W			2.061			

Bold values represent significance (*p* < 0.05)

#### Comparison of treatment subgroups

A subgroup analysis was performed to compare the macular structural and choroidal vascular parameters between patients receiving DFX monotherapy and those receiving combined DFX and DFO therapy. As detailed in Supplementary Table S3, there were no significant differences in the thickness of the central GCL, IPL, RNFL, GCC, or in the CVI between the two treatment groups (all *P* > 0.05). However, it is worth noting that the patients in the combined treatment group were significantly older, the duration of blood transfusion was significantly longer, and the SF level was also higher (all *P* < 0.01), indicating that their systemic iron load was more severe at baseline.

### Characteristic changes of the fundus

Characteristic ocular manifestations in TDT patients included: PXE in 4 eyes (6.56%), Tilted optic disc in 38 eyes (62.30%), Tessellated fundus in 21 eyes (34.43%), Retinal mottling in 15 eyes (24.56%), Peripheral ‘white without pressure’ lesions in 10 eyes (16.39%), Peripheral ‘black without pressure’ lesions in 32 eyes (52.46%), and Retinal pigment epithelial degeneration in 2 eyes (3.28%) (Table 6).

**Table 6:** The distribution of characteristic retinal changes in TDT patients

No.	TDT Group (*N* = 61)	*n* (%)
1	Pseudoxanthoma elasticum(PXE)	4 (6.56)
2	Tilted optic disc	38 (62.30)
3	Tessellated fundus	21 (34.43)
4	Mottled retinopathy	15 (24.56)
5	Peripheral ‘white without pressure’ lesions	10 (16.39)
6	Peripheral ‘black without pressure’ lesions	32 (52.46)
7	Retinal pigment epithelial degeneration	2 (3.28)

## Discussion

In this cross-sectional study, we systematically analyzed macular structure and blood flow density in a Southern Chinese TDT cohort using OCT/OCTA, revealing significant alterations in macular microstructure and microcirculation. Furthermore, we identified correlations between both mGCC thickness and CVI with systemic iron overload markers. These findings provide an important basis for the early monitoring of fundus lesions in patients with TDT.

Consistent with prior studies [14], TDT patients exhibited marked thinning of the GCL, GCC, and RNFL in the macular region in our study. The observed non-uniform thinning of the GCL occurred predominantly in the central, inner nasal, inner superior, inner temporal, and outer inferior sectors, but not in others. The macular GCL distribution follows a specific topographic pattern, with the highest density in the central and paracentral regions [20]. We speculate that this pattern could be due to regional variationsin retinal ganglion cell density [20], metabolic demand [21, 22], or microvascular architecture [23], which may render certain sectors more susceptible to the cumulative insult of iron-induced oxidative stress [8]. In fact, further studies with larger sample sizes are needed to validate this topographic pattern and elucidate its underlying mechanisms. Notably, the positive correlation between central mGCC thickness and ICT duration (*r* = 0.280, *P* < 0.05) suggested a neuroprotective effect of chelators by reducing retinal iron accumulation [24]. However, previous studies have shown the potential retinal toxicity of DFO and other chelators [25, 26] underscores the need for personalized dosing regimens to balance efficacy and ocular safety. This apparent contradiction highlights that the dosage and treatment course of iron chelating agents need to be individualized and optimized to balance the therapeutic effect of iron removal and the safety of the retina.

Our results showed that, although the macular area thinned, the blood flow densities of DVC, NFLVP and DCP increased significantly. We hypothesize that this seemingly contradictory phenomenon may be related to a compensatory mechanism triggered by chronic retinal hypoxia. As a highly oxygen-consuming tissue, the retina is exquisitely sensitive to hypoxia. Under such conditions, hypoxia-inducible factor-1α (HIF-1α) is stabilized and acts as a master transcriptional regulator, upregulating downstream targets like VEGF [27]. It is well documented that serum VEGF levels are elevated in patients with chronic hematological diseases [28]. Furthermore, beyond its role in angiogenesis, VEGF is a potent vasodilator that can directly increase blood flow by signaling through its VEGF receptor 2 [29]. Therefore, the increased flow density could be explained by VEGF-mediated vasodilation and increased flow velocity, representing a compensatory microvascular adjustment to maintain oxygen delivery. However, this interpretation remains speculative, as we did not measure serum or ocular VEGF levels to directly corroborate this hypothesis. It is also possible that this compensatory state is transient, and prolonged hypoxia coupled with direct iron toxicity could ultimately lead to vascular dropout in later disease stages—a possibility our cross-sectional design cannot capture. Future studies incorporating VEGF biomarkers are needed to validate this proposed hypothesis.

Furthermore, our research found that alterations in mGCC thickness and CVI could occur independently in some patients. This observation suggests that the retina and choroid may exhibit differential susceptibility or response pathways to systemic iron overload. The thinning of the mGCC, comprising the retinal nerve fiber, ganglion cell, and inner plexiform layers, is a recognized marker of neuronal loss, which in the context of iron overload, may be driven by oxidative stress-mediated damage to retinal ganglion cells [8, 30, 31]. In contrast, the elevation in CVI, reflecting a shift in the choroidal stroma-to-lumen ratio, was significantly correlated with systemic ferritin levels (*r* = 0.426, *P* < 0.01). Previous studies have shown that iron deposition may promote the remodeling of the choroid [32, 33]. This association points to CVI as a potential indicator of choroidal vascular remodeling linked to the body’s iron burden. Consequently, mGCC thickness and CVI appear to provide complementary information: the former quantifying structural neuronal injury, and the latter reflecting a vascular-structural adaptation of the choroid to iron overload. The simultaneous in vivo assessment of both parameters via OCT/OCTA offers a more comprehensive profile of the distinct ocular compartments affected in transfusion-dependent β-thalassemia.

Our subgroup analysis (Supplementary Table S3) revealed no significant differences in macular neuroretinal thickness or choroidal vascularity between patients on DFX monotherapy and combination therapy. This finding should be interpreted with caution, as the groups were not randomized. The patients assigned to combination therapy had a significantly higher pre-existing iron burden, as evidenced by longer transfusion duration and elevated SF. This clinical practice aligns with treatment guidelines, where combination therapy is often reserved for cases with more severe iron overload or inadequate response to monotherapy [34]. The microstructural changes captured by OCT/OCTA have a complex relationship with systemic iron metrics and are influenced by factors beyond the chelation regimen itself. Future prospective studies are warranted to delineate the specific ocular benefits of different iron chelation strategies.Furthermore, in our study, we also observed that the incidence of retinal pigment epithelium degeneration (3.28%) was significantly lower than the reported value in southern Iran (15.6%) [35]. Additionally, the occurrence frequency of tilted optic discs (62.30%) was higher than that in the general population [36]. This may be related to the potential genetic regulatory factors and regional genetic variations in TDT patients in southern China. Studies have shown that the prevalence of PXE worldwide is 1/56,000 to 1/25,000 [37]. We observed a significantly higher prevalence of PXE in the TDT population (6.56%). These patients have a higher risk of further retinal detachment and require enhanced monitoring.

This study has several limitations that should be considered when interpreting the results. First, its cross-sectional design precludes the establishment of causal relationships or temporal sequences between iron load and observed macular changes. Longitudinal studies incorporating ICT interventions are needed to evaluate temporal dynamics and treatment effects. Second, the moderate sample size from a single center may affect the generalizability of our findings. A post-hoc power analysis indicated that the key association between CVI and SF was robust (power >0.93). Similarly, comparisons for parameters like AL, GCC of the inner nasal and inner temporal, GCL of the inner nasal and inner temporal, NFLVP between TDT and control groups achieved a power >0.80. However, several other significant comparisons, particularly for specific macular layer thicknesses, were underpowered (power < 0.80). Therefore, these latter findings should be interpreted with caution and viewed as preliminary for future verification. Third, the lack of direct retinal or choroidal iron quantification (e.g., MRI T2* mapping) limits the depth of mechanistic insight regarding local iron deposition. Future work should combine multimodal imaging with serum/ocular biomarkers to establish predictive models for TDT-related retinopathy progression. Fourth, our study exclusively enrolled pediatric patients (4–18 years), which may limit the generalizability of our findings to adult TDT populations. Adult patients with longer disease duration might exhibit different or more advanced ocular manifestations [38]. Future studies should include adult cohorts to establish age-related progression patterns. Finally, the use of a 3 × 3 mm OCTA scan precludes the assessment of the peripheral retinal microvasculature. Consequently, our findings are specific to the central retina. Future wide-field OCTA studies would provide a more comprehensive topographic map of ocular involvement in TDT.

Notably, this study benefits from the regional representativeness of Guangdong Medical University Affiliated Hospital, the sole hematopoietic stem cell transplantation center in western Guangdong. We propose that alterations in DVC, NFLVP, and DCP blood flow density represent sensitive markers for early macular microvascular dysregulation in TDT, potentially useful for refining chelation therapeutic windows. The validation of CVI as a non-invasive biomarker for systemic iron overload severity holds promise for guiding personalized chelation strategies and monitoring their ocular effects.

## Conclusions

In this cross-sectional study, TDT patients demonstrated characteristic alterations including macular neuroretinal thinning (particularly in the mGCC) and increased microvascular blood flow density in specific deep retinal plexuses. CVI and central mGCC thickness metrics show promise as potential biomarkers for monitoring iron-related ocular pathology and therapeutic efficacy. However, their predictive value and causal relationships require validation through longitudinal studies. Regular OCT/OCTA screening, combined with hematologic profiling, should be integrated into TDT management protocols to optimize iron overload control and prevent vision-threatening complications. This study provides imaging-based evidence to advance early diagnosis and tailored therapeutic strategies for TDT-associated retinopathy.

## Supplementary Information

Below is the link to the electronic supplementary material.

Supplementary Material 1

## Data Availability

The datasets generated during and analysed during the current study are not available due to the protection of data security (the original data contains a lot of specifically demographic characteristics information and will be used again in the future follow-up study) but are available from the corresponding author on reasonable request.

## Abbreviations

TDT: Transfusion-dependent β-thalassemia
OCT: Optical coherence tomography
OCTA: Optical coherence tomography angiography
CVI: Choroidal vascular index
SF: Serum ferritin
ICT: Iron chelation therapy
mGCC: Macular ganglion cell complex
GCC: Ganglion cell complex
PXE: Pseudoxanthoma elasticum
RGC: Retinal ganglion cell
AL: Axial length
BMI: Body mass index
Hb: Hemoglobin
BCVA: Best-corrected visual acuity
IOP: Intraocular pressure
SER: Spherical equivalent refraction
ETDRS: Early treatment diabetic retinopathy study
EDI: Enhanced depth imaging
SVC: Superficial vascular complex
DVC: Deep vascular complex
NFLVP: Nerve fiber layer vascular plexus
SVP: Superficial vascular plexus
ICP: Intermediate capillary plexus
DCP: Deep capillary plexus
RNFL: Retinal nerve fiber layer
GCL: Ganglion cell layer
IPL: Inner plexiform layer
TCA: Total choroidal area
CLA: Choroidal luminal area
CSA: Choroidal stromal area
SD: Standard deviation
DFX: Deferasirox
DFO: Deferoxamine
VEGF: Vascular endothelial growth factor
RPE: Retinal pigment epithelium
