# Supplementary material for: Iron-associated central macular ganglion cell complex thinning and choroidal vascularity index elevation in transfusion-dependent β-thalassemia: potential OCT/OCTA biomarkers
Source: BMC Ophthalmol. 2025 Dec 12;25:690. doi: 10.1186/s12886-025-04510-0 (PMC12699819; doi:10.1186/s12886-025-04510-0)
Supplement: Supplementary file 1 — Supplementary Material 1 [file 12886_2025_4510_MOESM1_ESM.pdf]

Supplement

Supplementary Fig. Sa Schematic diagram illustrating the ETDRS grid segmentation method for macular structural analysis

Supplementary Fig. Sb Subfoveal choroidal area measurement Images were binarized using the Niblack method within ImageJ software (version 1.53; National Institutes of Health, Bethesda, MD, USA) and the ratio of the luminal area (hyporeflective) to the stromal area (hyperreflective) was quantified, with a measurement window width of 1500 μm positioned beneath the fovea

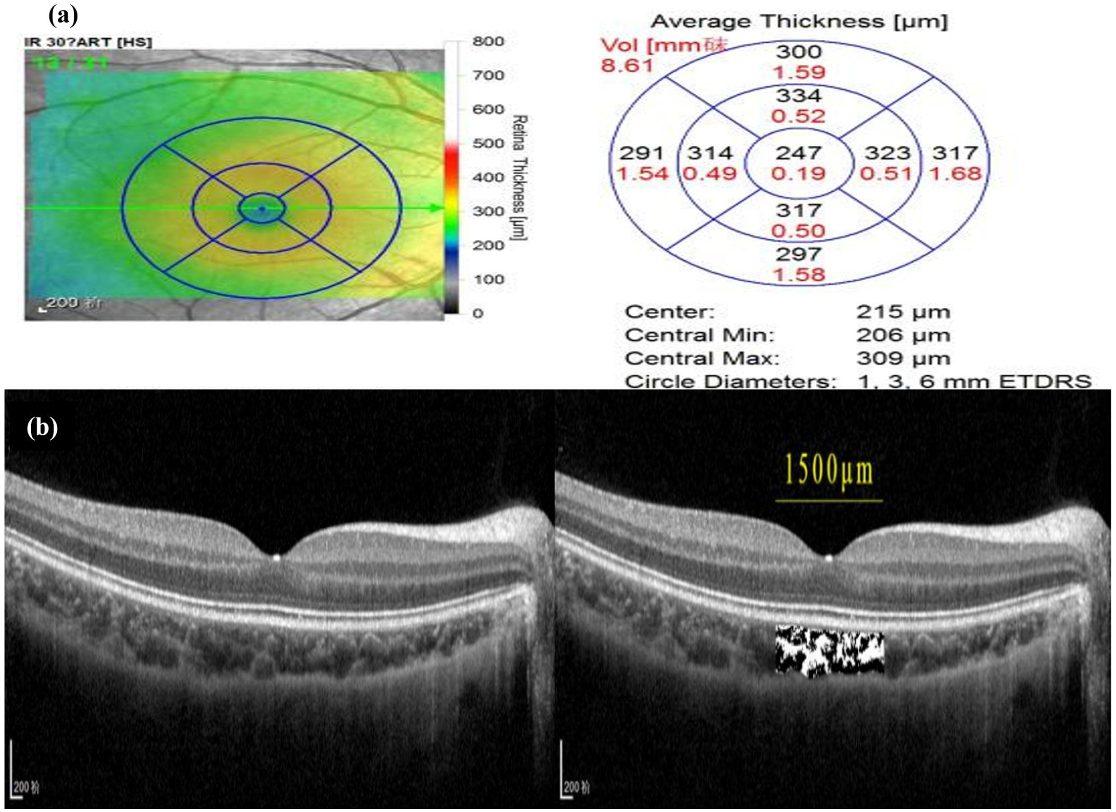

(a)
IR 30?ART [HS]
800
700
600
500
400
300
200
100
0
Retina Thickness [µm]
Average Thickness [µm]
Vol [mm
8.61
300
1.59
334
0.52
291
1.54
314
0.49
247
0.19
323
0.51
317
1.68
317
0.50
297
1.58
Center: 215 µm
Central Min: 206 µm
Central Max: 309 µm
Circle Diameters: 1, 3, 6 mm ETDRS
(b)
1500µm

Supplementary Table S1 Sociodemographic and clinical characteristics of the participants by groups

		TDT Group （N=61）	Control Group (N=61)	X^2/t	P [a]
		Mean ± SD / Median (IQR)			
Gender	Boy	37（60.66%）	31（50.82%）	1.196	0.274
	Girl	24（39.34%）	30（48.18%）		
SER (D), Mean ± SD		-0.39 ± 2.19	-0.72 ± 1.29	0.994	0.323
Age (ys), Mean ± SD		10.31 ± 3.67	11.00 ± 3.18	-1.106	0.271
AL (mm), Mean ± SD		23.32 ± 0.97	24.06 ± 0.99	-4.183	**<0.001**
BMI (kg/m^2), Mean ± SD		15.39 ± 1.46	-	-	-
Pre-transfusion Hb (g/L), Mean ± SD		80.45 ± 15.96	-	-	-
Transfusion Duration (ys), Mean ± SD		8.78 ± 3.79	-	-	-
FeHb (<1month) (ug/L), Median (IQR)		4070.00 (3024.00, 6270.00)	-	-	-
ICT Duration (ys), Mean ± SD		3.17 ± 2.54	-	-	-
Iron Chelator Type	1	31（50.82%）	-	-	-
	2	30（49.18%）			

N number

[a] Continuous variables were calculated by T-tests, and categorical variable were calculated by Chi-square test.

Bold values represent significance (p<0.05).

Supplementary Table S2 Analysis of the correlation factors of macular structural thickness, blood flow density and choroidal parameters in the TDT group

	IN GCL	IN GCC	IS GCL	IS GCC	IT GCL	IT GCC	OI GCL	Central GCL	Central RNFL	Central GCC	CSA	CVI	DCP	NFLVP	DVC
Gender	-0.054	-0.135	-0.014	-0.078	0.085	-0.044	0.143	-0.135	0.09	-0.105	-0.228	0.080	-0.017	0.088	0.001
SER (D)	-0.375 **	-0.400 **	-0.288 *	-0.292 *	-0.263 *	-0.270 *	-0.284*	0.312 *	-0.077	0.336 **	0.217	0.116	-0.037	-0.036	-0.072
Age (ys)	0.250	0.262 *	0.225	0.268 *	0.010	-0.046	-0.041	-0.060	-0.107	-0.105	-0.005	-0.171	-0.020	0.045	-0.017
AL (mm)	0.233	0.267 *	0.294 *	0.287 *	0.132	0.134	-0.066	0.004	0.034	-0.032	-0.025	-0.234	0.090	-0.111	0.061
BMI (kg/m^2)	0.035	0.087	0.045	0.140	-0.090	-0.079	-0.220	-0.039	-0.202	-0.071	0.201	-0.118	-0.102	-0.060	-0.105
Pre-transfusion Hb (g/L)	0.312 *	0.272 *	0.130	0.095	0.223	0.221	0.090	-0.208	0.011	-0.204	0.043	-0.049	0.200	0.201	0.229
Transfusion Duration (ys)	0.199	0.202	0.278 *	0.293 *	0.085	0.023	0.019	-0.116	-0.076	-0.143	-0.093	-0.177	-0.104	0.065	-0.096
SF (<1month) (ug/L)	0.046	-0.021	-0.008	-0.035	0.107	0.087	-0.025	-0.155	0.183	-0.128	-0.250	0.426 **	0.019	-0.111	0.010
ICT Duration (ys)	0.025	0.009	0.195	0.157	-0.139	-0.104	-0.197	0.278 *	0.028	0.280 *	0.151	-0.220	0.183	0.142	0.202
Iron Chelator Type (1/2)	0.142	0.133	-0.054	0.070	0.115	0.118	-0.042	0.002	0.030	0.000	0.128	-0.055	-0.134	0.084	-0.092

IN inner nasal, IS inner superior, IT inner temporal OI outer inferior

* p<0.05 and ** p<0.01were considered statistically significant.

Supplementary Table S3 Comparison of demographic, systemic iron overload, and ocular parameters between DFX monotherapy and DFX+DFO combination therapy subgroups in TDT patients

		DFX Group (N=31)	DFX+DFO Group (N=30)	*t/Z*	*P* [a]
		Mean ± SD / Median (IQR)			
Age (ys), Mean ± SD		8.88 ± 3.52	11.79 ± 3.25	-3.344	**<0.001**
Transfusion Duration (ys), Mean ± SD		7.41 ± 3.54	10.20 ± 3.556	-3.075	**0.003**
SF (<1month) (ug/L), Median (IQR)		3321.00 (1531.00, 4310.00)	6120.00 (3680.00, 9691.00)	-3.594	**<0.001**
CVI (%), Mean ± SD		66.71 ± 3.20	66.31 ± 4.22	0.422	0.674
Central, Median (IQR)	GCL	13.00 (10.00, 16.00)	14.00 (11.75, 16.25)	-1.052	0.293
	IPL	17.00 (10.00, 21.00)	17.00 (12.50, 20.25)	-0.297	0.767
	RNFL	12.00 (10.00, 15.00)	11.50 (10.00, 18.00)	-0.022	0.983
	GCC	41.00 (37.00, 48.00)	44.50 (40.00, 48.75)	-0.961	0.337

N number

[a] Continuous variables were calculated by T-tests or Non-parametric tests.

Bold values represent significance (p<0.05).
